# An Anaplastic Thyroid Carcinoma of the Giant-Cell Type from a Mediastinal Ectopic Thyroid Gland

**DOI:** 10.3390/diagnostics13182941

**Published:** 2023-09-14

**Authors:** Daniel Nguyen, Nyein Nyein Htun, Beverly Wang, Bonnie Lee, Cary Johnson

**Affiliations:** 1 Department of Pathology and Laboratory Medicine, University of California, Irvine, CA 92868, USA; nhtun@hs.uci.edu (N.N.H.); bevwang@hs.uci.edu (B.W.); bonniel6@hs.uci.edu (B.L.); caryj@hs.uci.edu (C.J.); 2 Department of Dermatology, University of California, Irvine, CA 92868, USA

**Keywords:** ectopic thyroid, mediastinal mass, anaplastic thyroid carcinoma, giant-cell thyroid carcinoma, interesting images

## Abstract

Anaplastic thyroid carcinoma is a rare, aggressive form of thyroid carcinoma with a mean survival of less than 6 months. Ectopic thyroid tissue can be present in the mediastinum due to faulty embryogenesis with improper descent. Primary thyroid malignancies may arise from this ectopic tissue. A 90-year-old male with a history of prostatic adenocarcinoma, hypothyroidism, and occupational and therapeutic exposure to radiation presented with a rash on his chest. A review of the dermatopathology and excised mediastinal specimen revealed rare papillary foci that tested positive for thyroid markers from a background of poorly differentiated components. Molecular analysis confirmed a BRAF V600E mutation in the specimen. The final diagnosis was anaplastic thyroid carcinoma of the giant-cell type. Given the atrophic cervical thyroid tissue in the patient’s neck with no evidence of previous surgery, this carcinoma was believed to arise from ectopic mediastinal tissue associated with cutaneous and bony metastasis. In conclusion, anaplastic thyroid carcinoma is an aggressive and rare thyroid malignancy that can arise from ectopic thyroid tissue in the mediastinum and should be considered in the differential diagnosis of primary undifferentiated mediastinal malignancies with bony involvement.

The patient examined was a 90-year-old with a medical history of hypothyroidism treated with levothyroxine and prostatic adenocarcinoma treated with radioactive seeds 20 years prior to the examination. The patient exhibited multiple raised skin lesions of up to 10 cm on his chest and weight loss amounting to 30 pounds. Computed tomography of the chest identified a large expansile soft tissue mass believed to arise from the sternal manubrium and the upper to mid-sternal body. The mass measured 12 cm in length, had a cross-sectional diameter of 11 cm, and infiltrated adjacent soft tissue structures and anterior mediastinal fat (Figure 1A). Given the possibility of the role of the thyroid in the differential diagnosis, a review of the roentgenogram was performed, which showed the absence of an atrophic gland (Figure 1B) without evidence of previous surgery. 

The skin lesion was biopsied. Two separate components were microscopically identified: a rare glandular focus (Figure 2A) and a second poorly differentiated component (Figure 2B). The glandular focus was papillary and positive for AE1/AE3, TTF1, and PAX8 (Figure 2C–E). CD68 was positive in scattered giant cells in the poorly differentiated focus (Figure 2F). Since a thyroid gland was absent, an anaplastic thyroid carcinoma caused by ectopic thyroid was the main differential diagnosis. Ectopic thyroid is a developmental abnormality where the thyroid gland fails to migrate from the primitive foregut floor to the anterior neck. Ectopic thyroid may result in hypothyroidism, hyperthyroidism, or no clinical symptoms [1]. Ectopic thyroid tissue developing malignancy is relatively rare, occurring in less than 1% of cases [2]. Multiple termini have been reported, including the lateral side of the neck, the sternocleidomastoid muscle, and the tongue. The most common ectopic cancer type is papillary thyroid carcinoma, which is consistent with the prevalence of papillary thyroid carcinoma in the thyroid [3,4,5]. An excisional biopsy of the mediastinal mass was performed to confirm the diagnosis.

The carcinoma’s histopathology consisted primarily of a poorly differentiated tumor with abundant giant cells. Rare minute papillary foci were identified in addition to a poorly differentiated component on the single frozen section slide (Figure 3A,B). TTF1, thyroglobulin, PAX8, and BRAF V600E were all positive (Figure 3C–F). SATB2 and CD68 were positive in scattered giant cells (Figure 3G,H). A diagnosis of anaplastic thyroid carcinoma with giant cells was made. Anaplastic thyroid carcinoma is a relatively uncommon carcinoma. The corresponding prognosis is very poor, with an average median relative survival of 3 to 6 months [6]. Histologically, anaplastic thyroid carcinoma may present with multiple histomorphological variants, such as pleomorphic, epithelioid, spindle cell, rhabdoid, osteoclast giant-cell-rich, and squamous cell carcinoma [7]. Due to its variable morphological presentation, immunohistochemistry is required for diagnosis. The literature reports strong staining results with AE1/AE3, CAM5.2, P53, and PAX-8 positivity in 36% of cases. TTF-1 and thyroglobulin, thyroid lineage markers, are usually negative [8]. Giant-cell-rich anaplastic thyroid carcinoma is a rare pattern within the anaplastic thyroid carcinoma morphological spectrum, with only a few cases reported in the literature. Histologically, numerous osteoclast giant cells with multiple nuclei and elevated mitotic activity have been reported [9]. The corresponding immunohistochemical staining patterns are like those of other types of anaplastic thyroid carcinoma with a negative expression of TTF-1 and thyroglobulin. Common mutations in anaplastic thyroid carcinoma include TERT promoter mutation (73%), TP53 (59%), BRAF (29%), and RAS (23%) [10]. Additional immunohistochemical staining procedures were performed to exclude other entities in the differential diagnosis, such as sarcomatoid carcinoma of the lung, prostate carcinoma, colon carcinoma, leiomyosarcoma, rhabdomyosarcoma, melanoma, dedifferentiated liposarcoma, angiosarcoma, myeloid sarcoma, and follicular dendritic cell sarcoma.

PET/CT of the whole body was performed to show how the neoplasm had grown to 15.2 cm (12 cm in the previous CT scan one month earlier, Figure 4A). Cervical and axillary lymphadenopathy was also observed (Figure 4B). The specimen was sent for molecular testing, which confirmed that the tumor harbored the BRAF V600E mutation. The patient died approximately six months after the primary pathological diagnosis.

In conclusion, anaplastic thyroid carcinoma is an aggressive and rare thyroid malignancy that can arise from ectopic thyroid tissue in the mediastinum and should be considered in the differential diagnosis of primary undifferentiated mediastinal malignancies with bony involvement.

## Figures and Tables

**Figure 1:**
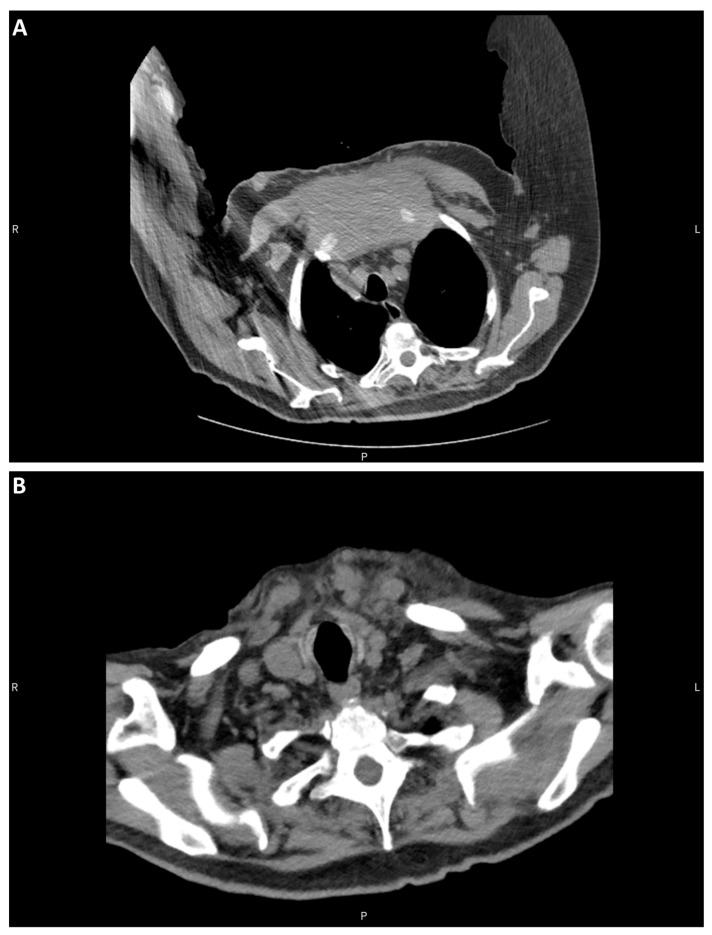
CT of the chest and neck. (**A**) Sternal mass. (**B**) Atrophic thyroid bed.

**Figure 2:**
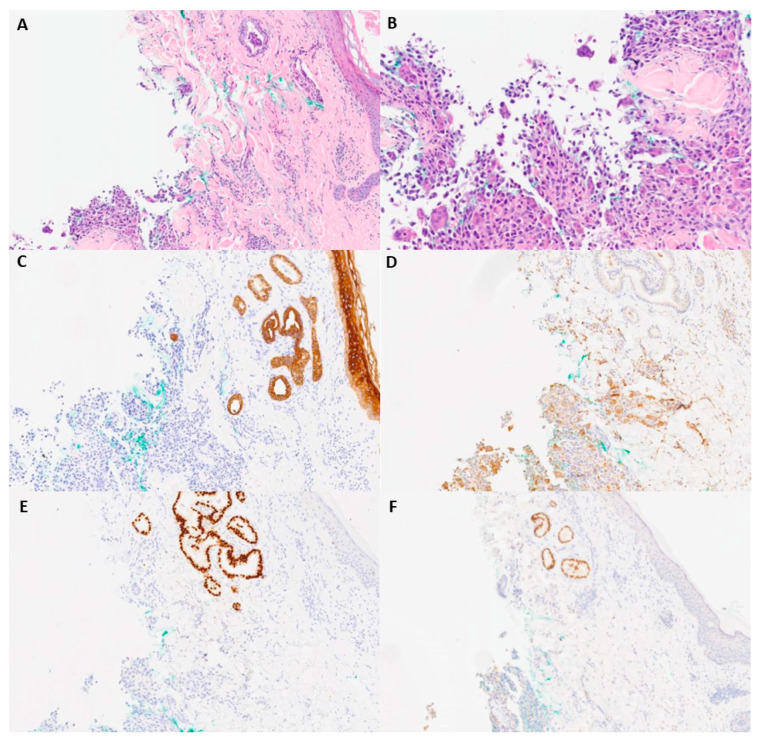
Microscopic and immunohistochemical examination of skin biopsy. (**A**) H&E-staining (40×) image with foci of papillary and multinucleated giant-cell neoplasms. (**B**) Higher-power H&E-staining image (100×) of poorly differentiated neoplasm foci. (**C**) AE1/AE3 (40×). (**D**) CD68 (40×). (**E**) TTF-1 (40×). (**F**) PAX-8 (40×).

**Figure 3:**
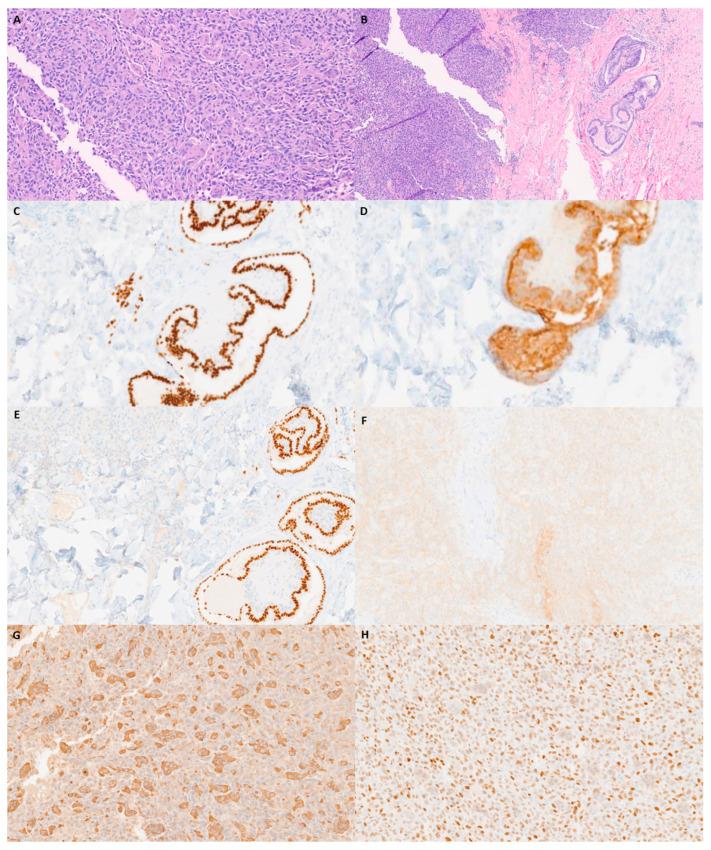
Microscopic and immunohistochemical examination of the sternal mass. (**A**) H&E (100×). (**B**) H&E with thyroid papillary foci (40×). (**C**) TTF-1 at papillary foci (100×). (**D**) Thyroglobulin in anaplastic tumor cells (100×). (**E**) PAX-8 in anaplastic tumor cells (100×). (**F**) BRAF V600E (100×). (**G**) CD 68 (100×). (**H**) SATB2 (100×).

**Figure 4:**
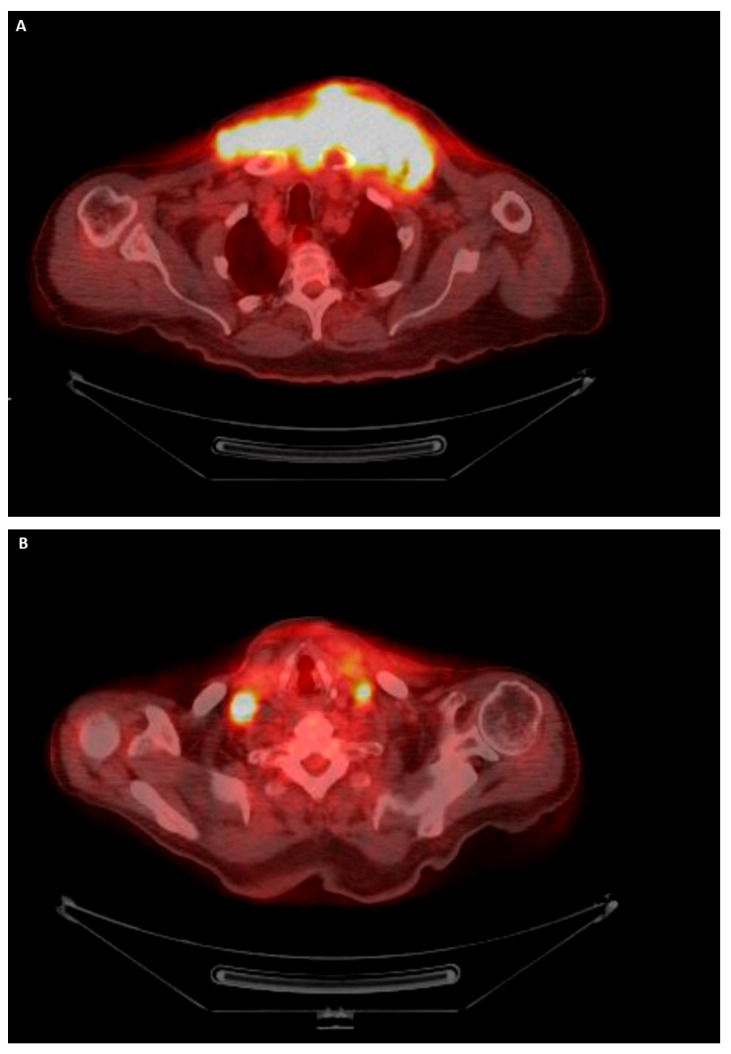
PET/CT imaging of the whole of the body. (**A**) Mediastinal mass. (**B**) Lymphadenopathy.

## Data Availability

Not applicable.
